# Fatty Fish Intake and the Effect on Mental Health and Sleep in Preschool Children in FINS-KIDS, a Randomized Controlled Trial

**DOI:** 10.3390/nu10101478

**Published:** 2018-10-11

**Authors:** Mari Hysing, Ingrid Kvestad, Marian Kjellevold, Lisa Kolden Midtbø, Ingvild Eide Graff, Øyvind Lie, Hallvard Hurum, Kjell Morten Stormark, Jannike Øyen

**Affiliations:** 1 Regional Centre for Child and Youth Mental Health, NORCE Norwegian Research Centre, 5008 Bergen, Norway; Ingrid.kvestad@norceresearch.no (I.K.); hhurum@gmail.com (H.H.); kjst@norceresearch.no (K.M.S.); 2 Department of Psychosocial Science, Faculty of Psychology, University of Bergen, 5020 Bergen, Norway; 3 Institute of Marine Research (IMR), 5817 Bergen, Norway; marian.kjellevold@hi.no (M.K.); lisakolden.midtbo@hi.no (L.K.M.); ingr@norceresearch.no (I.E.G.); oyvind.lie@fiskeridir.no (Ø.L.); jannike.oyen@hi.no (J.Ø.); 4 NORCE Norwegian Research Centre, 5008 Bergen, Norway; 5 Directorate of Fisheries, 5804 Bergen, Norway; 6 Department of Health Promotion and Development, University of Bergen, 5020 Bergen, Norway

**Keywords:** docosahexaenoic acid, eicosapentaenoic acid, fatty fish, preschoolers, mental health, sleep

## Abstract

Mental health and sleep problems are prevalent in children during preschool years. The aim of the current study was to investigate if increased intake of fatty fish compared with meat improves mental health and sleep in four- to six-year-old children. The children (*n* = 232) in the two-armed randomized controlled trial, Fish Intervention Studies-KIDS (FINS-KIDS), were randomly assigned to lunch meals with fatty fish (herring/mackerel) or meat (chicken/lamb/beef) three times a week for 16 weeks. The fish and meat were weighed before and after the meals to record the exact consumption in grams (dietary compliance). Mental health problems were assessed by the strengths and difficulties questionnaire (SDQ) and sleep by parent report pre- and post-intervention. There was no significant statistical difference between changes in mental health and sleep for the fish eating group compared with the meat eating group, neither in the crude analysis nor after adjusting for intake of fish or meat (dietary compliance).

## 1. Introduction

Mental health and sleep problems are prevalent in children during preschool years [1,2]. Universal prevention programs are called for to improve mental health and sleep quality, and nutritional interventions may be one viable universal intervention method in young children. There has been a large interest in the potential health benefits of *n*-3 long-chain polyunsaturated fatty acids (*n*-3 LC-PUFAs) [3]. Because of the high levels of *n*-3 LC-PUFAs, increasing the intake of fatty fish may be beneficial. The fatty acids eicosapentaenoic (EPA), docosahexaenoic (DHA), and alpha linolenic (ALA) play a central role in brain development and functioning, and are central in the production of serotonin, which is important for both sleep and mental health [4,5]. Fatty fish is also relatively high in vitamin D, and the vitamin D status has been positively associated with sleep efficiency [6]. The *n*-3 LC-PUFAs have been linked to mental health in adults and elderly individuals [7,8]. Intervention studies have also been conducted, but most have included children in high-risk groups. The results from a meta-analysis concluded that omega-3 supplementation was modestly effective in children with ADHD [9] and aggression [10]. However, little is known regarding whether these effects are generalizable to a general population of preschool children.

Even less is known on the association between fish intake and sleep. Dietary fish intake has been associated with sleep quality in adult population studies [11], and the beneficial effect of fatty fish intake on sleep quality was confirmed in an intervention study, including a study involving adults in prison [6]. In neonates, an observational study has shown an association between prenatal DHA status and sleep patterns [12]. To our knowledge, there is only one randomized controlled trial (RCT) that includes sleep as a primary outcome. This RCT investigated the impact of DHA supplementation on sleep, and found conflicting results with a positive effect in a subsample with actigraphy measured sleep, but no effect of the supplementation on the questionnaire-based sleep assessment in the total sample [13]. Sleep and mental health are closely interrelated in preschool years [14], and thus including both sleep and mental health may allow for investigating the joint and independent outcomes.

Based on these considerations, the aim of the present study was to investigate the effect of fatty fish on mental health and sleep in a community-based individual RCT in preschoolers.

## 2. Materials and Methods

### 2.1. Study Design

The Fish Intervention Studies-KIDS (FINS-KIDS) is an RCT with two arms. Preschoolers were individually randomized to receive three warm lunch meals per week containing either fatty fish or meat for 16 weeks.

Seventeen out of the total 250 kindergartens in Bergen municipality were invited, and 13 agreed to participate. The participating kindergartens were from different districts in the municipality to ensure participations across socioeconomic groups. Children between four and six years old in the participating kindergartens with sufficient understanding of the Norwegian language to undergo cognitive testing, and where caregivers had sufficient language skills to complete questionnaires in Norwegian, were invited to participate. Exclusion criteria were any known food allergies.

The primary outcome of the study was to observe a change in scores on cognitive function from pre- to post-intervention, and the results are presented elsewhere [15]. In the current study, we present the secondary outcomes of the main study—mental health and sleep.

### 2.2. Procedure

The trial took place between January and June 2015. The inclusion and pre-intervention assessment were done during a six-week period.

The intervention started within one week after the pre-intervention assessment, and the post-study assessment started within one week after the last study meal was consumed. Pre- and post-intervention testing included blood samples, cognitive tests of the children, and questionnaires completed by the caregivers. A catering company (Søtt + Salt A/S, Bergen) prepared and delivered the study meals to each kindergarten. Each meal contained 50–80 g of either fatty fish (herring/mackerel) or meat (chicken/lamb/beef). A variety of identical side dishes was provided for both intervention groups with each meal. An active comparison group (meat) was chosen to enable control of the intake in the comparison group and to ensure that the supply of nutrients was different between the groups.

Research assistants were trained to serve the meals, and weighed the fish and meat before and after the lunch to record the exact consumption in grams for each participant. The exact consumption in each meal was summed up to a total amount, constituting dietary compliance for each child. Identical digital weights (Digital Glass Kitchen Scale, Soehnle, Nassau, Germany) were used in every kindergarten. The research assistants also recorded when children were absent. During the meals, kindergarten personnel were present and ensured that the children only consumed the foods from their own meal.

The mean number of study meals served to each child during the intervention period was 44.0 (SD 4.0), and the children in the meat group had a higher mean total intake of meat (2679 (872) g) than the children in the fish group had of fish (2070 (957) g) (*p* < 0.0001).

Biological samples included analyses of fatty acids in red blood cells (RBC); serum 25-hydroxyvitamin D_3_ (s-25(OH)D_3_), urinary iodine concentration (UIC), and s-ferritin. Omega-3 index was calculated and includes the content of EPA and DHA expressed as percentages of total fatty acids.

Parents completed an online questionnaire that included children’s weight, height, age and gender, parental education and family income, and information on children’s mental health and sleep characteristics.

The childrens’ food intake over the previous three months was assessed by a revised version of a validated food frequency questionnaire (FFQ) [16,17,18]. The caregivers were specifically instructed not to include the meals served in the kindergarten. In total, 49% of the children adhered to the recommendations for fish intake before the study. There were no differences in food intake within or between the groups, except for a slightly lower intake of red meat in the fish group, from pre- to post-intervention [15].

### 2.3. Outcome Measures

Mental health was assessed by the Strengths and Difficulties Questionnaire (SDQ). SDQ is a short screening tool measuring mental health in the last six months in children and adolescents [19]. SDQ consists of 25 items scored on a three-point scale, which is divided into five subscales—emotional problems, conduct problems, hyperactivity/inattention, peer relationship problems, and prosocial behavior. The total problem score is based on the sum of 20 items from all subscales, except for the subscale measuring pro-social behaviors. High scorers were defined by scores over the 80th percentile. The psychometric properties of the SDQ have been found to be strong [20,21]. The parental reported follow-up version of SDQ that was used post-intervention focused on symptoms during the previous four months (the study period).

Sleep was measured by parental questionnaire. Self-reported bedtime and rise time were indicated in hours and minutes using a scroll down menu with fifteen-minute intervals, and were reported separately for weekend and weekdays. Time in bed (TIB) was calculated by subtracting bedtime from rise time. Sleep onset latency (SOL) and wake after sleep onset (WASO) were indicated in hours and minutes using a scroll down menu with five-minute intervals. Sleep duration was defined as TIB minus SOL and WASO. Sleep efficiency was calculated as sleep duration divided by TIB multiplied by 100 (reported as a percentage).

### 2.4. Biochemical Analyses

The blood sampling was done in each kindergarten by two biomedical scientists blinded to the treatment conditions. Venous blood was collected in BD Vacutainer^®^ K2E 7.2 mg vials for preparation of RBC and BD Vacutainer^®^ SST^TM^ II Advance for preparation of serum, and centrifuged (10 minutes/1000 g/20 °C) within 30 minutes of sampling. They were then transferred to Cryptube (Nunc/Roskilde/Denmark) and transported on dry ice to storage at −80 °C until analysis. Mixed pre- and post-intervention samples were analysed after the intervention.

Fatty acid composition of total RBC was determined by standardized procedures at the Institute of Marine Research (IMR) [22] by ultrafast gas chromatographic (UFGC) (Thermo Electron Corporation, MA, USA).

S-25(OH)D concentration was determined by standardized procedures at IMR using a liquid chromatographic-tandem mass spectrometric (LC-MS/MS) assay, adding acetonitrile and internal standard (^2^H 25OH vitamin D_3_) to the samples.

S-ferritin was analysed at Haraldsplass Diakonale Hospital, Bergen, Norway by an automated electrochemiluminescence immunoassay (ECLIA) on Cobas e601 (Roche).

UIC was determined in spot samples by inductive coupled plasma mass spectrometry (ICP-MS) by standardized procedures at IMR [23].

### 2.5. Ethics

The study was approved by the Regional Committees for Medical and Health Research Ethics North (2014/1396), and is registered in ClinicalTrials.gov (NCT02331667). Written informed consent was obtained from the participants’ caregivers. Participants could withdraw from the study at any time.

The food safety aspects of the intervention have been evaluated and published elsewhere [15].

### 2.6. Statistical Analyses

The sample size calculation was based on cognitive scores, as this was the main outcome of this study, and is presented previously [15].

Continuous variables are expressed as mean and standard deviation (SD), and categorical variables as numbers and percentages. We used independent sample *t*-tests to compare intervention groups at baseline, changes from pre- to post-intervention on biochemical variables, and the intake of fish and meat (dietary compliance). A paired-samples *t*-test was used for analyses of differences between pre- and post-intervention mental health (SDQ) total and subscales scores, as well as sleep variables within each intervention group. Linear mixed effect models with random intercept for kindergartens were used to analyze changes (post- to pre-intervention) in mental health and sleep variables. First, the models were adjusted for pre-intervention scores, and secondly dietary compliance was included. In sub-analyses, an 80th percentile cut-off point for the SDQ was used to define the participants that scored high on the SDQ total difficulties.

Two-tailed *p* < 0.05 tests were considered statistically significant. Statistical analyses were performed using Statistical Package for the Social Sciences (SPSS^®^ Statistics Version 24 (IBM Corp., Armonk, NY, USA)).

## 3. Results

### 3.1. Study Population

**Figure 1:**
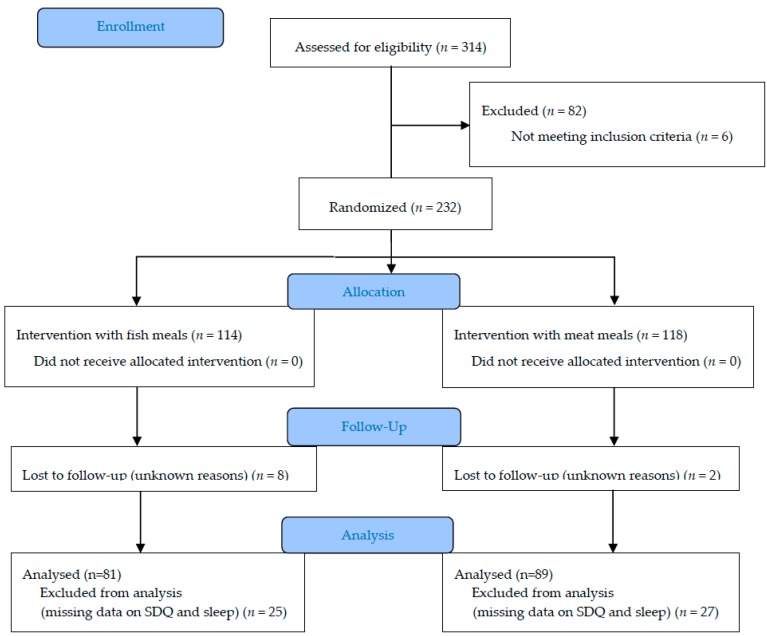
Overview of the study population. SDQ, Strengths and Difficulties Questionnaire.

In total, 222 children completed the intervention, of which 52 were excluded from the analyses because of missing data on SDQ or sleep. Thus, 170 (76.6%) children were included in the final study sample, and 81 (47.9%) and 89 (52.7%) in the fish and meat group, respectively (Figure 1). There were no significant differences between those who completed the intervention on baseline characteristics versus those who did not, and there were no differences between the study groups in the baseline characteristics. The characteristics at baseline are presented in Table 1. The mean (SD) age of the children was 5.2 (0.6) years and 91 (53.8%) were girls. The mean s-25(OH)D_3_ and omega-3 index was 61.4 (14.3) nmol/L and 7.4 (1.4)%, respectively. Data from FFQ show that the children consumed 1.7 (0.9) meals of fish, 2.5 (0.9) meals of red meat, and 1.3 (0.9) meals of chicken weekly before the intervention (Table 1).

### 3.2. Biochemical Parameters

Compared with the meat group, children in the fish had a significantly higher increase in the mean (SD) levels of EPA (0.02 (0.01)–0.03 (0.01) mg/g vs. 0.02 (0.01)–0.02 (0.01) mg/g, *p* < 0.001) and DHA (0.14 (0.03)–0.16 (0.04) mg/g vs. 0.14 (0.03)–0.14 (0.03) mg/g, *p* = 0.017) from pre- to post-intervention, respectively. Similar findings were observed for the omega-3 index (7.4 (1.4)–8.2 (1.6)% vs. 7.3 (1.4)–7.3 (1.5)%, *p* < 0.001). There was no significant difference in change of serum s-25(OH)D_3_ and UIC between the fish and meat group. S-ferritin increased more in the meat group than the fish group (27.5 (15.6)–29.2 (14.6) µg/L vs. 32.4 (19.9)–26.9 (10.7) µg/L, *p* = 0.001).

### 3.3. Mental Health

The pre- and post-intervention mean of the SDQ total and SDQ subscale scores are presented in Table 2. Crude analyses of change and models adjusting for pre-score, as well as a final model adjusting for pre-scores and dietary compliance, are included. There were no statistically significant differences between the fish and the meat intervention on the total or any of the subscale scores on the SDQ.

The sub analyses, including only the high scorers (above the 80th percentile), yielded no significant differences between groups.

### 3.4. Sleep

The mean level of sleep duration, SOL, and WASO pre- and post-intervention are presented in Table 3. Crude analyses of change and models adjusting for pre-score, as well as a final model adjusting for pre-scores and dietary compliance, are included. There were no statistically significant differences between the fish and the meat group on any of the included sleep measures.

## 4. Discussion

In this RCT in preschool children, there were no differences in mental health or sleep between children eating fatty fish compared with those eating meat for lunch three times per week for four months.

Although the intervention improved biological parameters including DHA and EPA in the fatty fish group, and the intervention has shown improvements in cognitive function when adjusting for dietary intake [15], the intervention showed no beneficial effects on mental health and sleep in any of the groups.

This is, to our knowledge, the first intervention in a general population and non-clinical sample of preschool children that investigates the effect of fatty fish in comparison with meat on mental health and sleep. Thus, comparisons to other studies are limited. Still, the lack of improvements in mental health problems in this population of preschool children did not support the promising findings of interventions in children with mental health problems [9,10]. This may be because of previous studies recruiting children with mental health problems and sleep problems, or children with nutritional deficiencies, specifically. The use of an active control group gives support to conclusions regarding the effect of a fish intervention in comparison with a meat intervention, but conclusions regarding the effect in comparison with regular meals is not possible with the lack of a blank control condition.

The biochemical analyses demonstrated that the fish intervention for 16 weeks was sufficient to improve the biological markers DHA and EPA, but not s-25(OH)D_3_ in comparison with meat intervention [15]. Still, the biochemical analyses show that these children have an adequate nutritional status and a high EPA and DHA level at baseline, compared with other children at a similar age [24]. Thus, the potential for further improvement might be limited. In comparison, the adult prison study, which is one of the few previous fish interventions with sleep as an outcome, had a six-month intervention period [6]. The intervention period in the present study could be too short for improvements in areas such as mental health and sleep.

The results need to be interpreted in light of the following limitations. The lack of change could be a result of no effect of the intervention on the domains of sleep and mental health, but it could also be a result of methodological limitations. Sleep was measured by parent report and not by objective indicators of sleep, and only sleep duration, SOL, and WASO for weekdays were included. This is in line with a previous intervention study that only found an effect on a subgroup assessed by actigraphy and not on the parent report [13]. However, previous research has shown a high correlation (*r* = 0.90) between parent-reported sleep duration and actigraphy-recorded sleep duration in young children, although with a slight overestimation of sleep duration from parents on the self-report supporting the assessment method [25]. Sleep during the weekdays will be impacted by the parents’ work schedule and daily routines. While this may have restricted the variability in sleep duration, which is a limitation for the study, this also reflected the environment that will be present during future universal interventions in similar settings. Similarly, mental health was assessed by a brief parent-reported screening instrument. Although it has shown good psychometric properties [21], it is mainly constructed for screening for problems, and may not be sensitive enough to detect differences in mental health over time. Further, in the present study, most children had good mental health and sufficient sleep, and the room for improvement on these outcomes was thus restricted. This may have led to lack of statistical power to detect differences.

The strengths of this trial include the RCT design, the close monitoring of fish or meat consumption, the low attrition, and the inclusion of biological markers of the biological mechanisms.

## 5. Conclusions

The presents study did not find changes in sleep and mental health in a general population of children. This is the first study of its kind and more studies, preferably with more statistical power, should be conducted before firm conclusions regarding universal prevention intervention for mental health and sleep in preschool children with good nutritional status are taken. There is also a need for studies in high risk children that can assess the possible impact of indicative intervention. Thus, future studies should include high risk groups both in terms of impaired mental health and sleep, and in terms of nutritional deficiencies, and include broader and improved outcome measures before we can conclude on the possible beneficial effect of fatty fish on mental health and sleep in preschool children.

## Figures and Tables

**Table 1:** Characteristics of the participants at baseline ^1^.

	*N*	Fish Group (*n* = 81)	Meat Group (*n* = 89)
Demographics			
Age, years	169	5.2 (0.6)	5.2 (0.5)
Body weight, kg	157	20.0 (3.4)	20.3 (2.9)
Body height, cm	159	114.1 (6.2)	113.8 (6.0)
Boys, *n* (%)	79	37 (45.7)	42 (47.2)
Girls, *n* (%)	91	44 (54.3)	47 (52.8)
Family income in NOK, *n* (%)			
<200,000–749,999	43	21 (26.3)	22 (24.7)
750,000–1,249,999	99	43 (53.8)	56 (62.9)
1,250,000–>2,000,000	27	16 (20.0)	11 (12.4)
Education parents, years	169	15.5 (1.6)	15.4 (1.6)
Sleep			
TIB, hour/min	169	11.23 (0.37)	11.18 (0.29)
Sleep duration, hour/min	169	10.55 (0.40)	10.45 (0.39)
WASO, minute/second	169	4.3 (8.6)	5.6 (10.5)
SOL, minute/second	169	23.9 (15.9)	27.4 (19.2)
SDQ			
Emotional problems	169	1.4 (1.4)	1.2 (1.3)
Conduct problems	169	1.3 (1.2)	1.4 (1.2)
Hyperactivity/inattention	169	2.3 (2.1)	2.4 (2.0)
Peer problems	169	0.8 (1.2)	0.9 (1.2)
Total problems	169	5.9 (4.0)	5.8 (3.8)
Biological parameters			
25(OH)D_3_, nmol/L serum	158	61.9 (14.5)	60.9 (14.1)
Ferritin, µg/L serum	162	32.4 (19.9)	27.5 (15.6)
EPA, 20:5 *n*-3, % of fatty acids in RBC	160	0.91 (0.43)	0.90 (0.35)
DHA, 22:6 *n*-3, % of fatty acids in RBC	160	6.7 (1.1)	6.4 (1.1)
Omega-3 index ^2^	160	7.4 (1.4)	7.3 (1.4)
UIC, µg/L urine	163	151.3 (96.1)	157.2 (98.6)
Dietary intake from FFQ, meals/week			
Fish as dinner	169	1.8 (0.9)	1.6 (0.9)
Red meat as dinner	169	2.6 (0.8)	2.4 (1.0)
Chicken as dinner	169	1.4 (0.9)	1.1 (0.9)
Fish as spread	169	1.5 (1.5)	1.2 (1.3)
*n*-3 LC-PUFA supplements, *n* (%)	74	29 (35.8)	37 (41.6)
No *n*-3 LC-PUFA supplements, *n* (%)	123	52 (64.2)	52 (58.4)

^1^ Values are means (SD) unless otherwise noted. ^2^ The content of EPA and DHA expressed as percentages of total fatty acids. There were no significant differences between the study groups (*p* > 0.05). Abbreviations: FFQ, food frequency questionnaire; DHA, docosahexaenoic acids; EPA, eicosapentaenoic acids; NOK, Norwegian kroner; *n*-3 LC-PUFA, long chain omega-3. fatty acids; RBC, red blood cells; SDQ, strengths and difficulties questionnaire; s-25(OH)D_3_, serum 25-hydroxyvitamin D_3_; SOL, sleep onset Laency, TIB, time in bed; WASO, wake after sleep onset; UIC, urinary iodine concentration.

**Table 2:** Predicted changes in parent-reported mental health measured by the Strengths and Difficulties Questionnaire (SDQ) total and subscales scores after fish (*n* = 81) or meat (*n* = 89) intervention.

	Crude			Models Adjusted For			
				Pre-Score		Pre-Score, Dietary Compliance	
SDQ	Pre Mean (SD)	Post Mean (SD)	*p*-Value ^1^	Change Mean (95% CI)	*p*-Value ^2^	Change Mean (95% CI)	*p*-Value ^3^
Emotional problems							
Fish	1.4 (1.4)	1.3 (1.5)	0.652	−0.02 (−0.29, 0.24)	0.765	0.02 (−0.03, 0.28)	0.505
Meat	1.2 (1.3)	1.1 (1.5)	0.749	−0.08 (−0.33, 0.17)		−0.11 (−0.37, 0.14)	
Conduct problems							
Fish	1.3 (1.2)	1.3 (1.3)	0.641	0.04 (−0.22, 0.30)	0.501	0.05 (−0.22, 0.31)	0.480
Meat	1.4 (1.2)	1.3 (1.2)	0.404	−0.07 (−0.32, 0.18)		−0.08 (−0.33, 0.18)	
Hyperactivity/inattention							
Fish	2.3 (2.1)	2.6 (2.3)	0.610	0.10 (−0.23, 0.42)	0.536	0.09 (−0.25, 0.42)	0.640
Meat	2.4 (2.0)	2.3 (2.0)	0.880	−0.03 (−0.35, 0.28)		−0.02 (−0.35, 0.31)	
Peer problems							
Fish	0.8 (1.2)	0.9 (1.2)	0.401	0.07 (−0.15, 0.29)	0.135	0.11 (−0.12, 0.34)	0.064
Meat	0.9 (1.2)	0.7 (1.1)	0.135	−0.16 (−0.37, 0.05)		−0.19 (−0.41, 9.02)	
Total problems							
Fish	5.9 (4.0)	6.1 (4.6)	0.573	0.22 (−0.47, 0.91)	0.191	0.29 (−0.41, 0.99)	0.127
Meat	5.8 (3.8)	5.4 (3.8)	0.256	−0.37 (−1.03, 0.30)		−0.44 (−1.11, 0.24)	

^1^ Paired-samples *t*-test for comparison of individual pre- and post-intervention values within each intervention group. ^2^ Linear mixed effect model adjusted for pre-intervention score. ^3^ Linear mixed effect model adjusted for pre-intervention score and dietary compliance (amount of fish/meat consumed).

**Table 3:** Predicted change in parent-reported sleep parameters after fish (*n* = 81) or meat (*n* = 89) intervention.

	Crude			Models Adjusted For			
				Pre-Score		Pre-Score, Dietary Compliance	
Sleep Parameters (minutes)	Pre Mean (SD)	Post Mean (SD)	*p*-Value ^1^	Change Mean (95% CI)	*p*-Value ^2^	Change Mean (95% CI)	*p*-Value ^3^
TIB							
Fish	683 (37)	678 (42)	0.075	−5.0 (−11.1, 1.0)	0.779	−5.8 (−11.8, 0.3)	0.905
Meat	678 (29)	672 (34)	0.033	−6.1 (−12.0, −0.2)		−5.3 (−11.2, 0.6)	
Sleep duration							
Fish	655 (40)	653 (45)	0.592	−0.4 (−7.5, 6.6)	0.614	−1.2 (−8.3, 5.9)	0.893
Meat	645 (39)	644 (36)	0.761	−2.6 (−9.5, 4.3)		−1.8 (−8.8, 5.1)	
WASO							
Fish	3.7 (8.0)	1.3 (4.7)	<0.001	−3.3 (−4.1, −2.5)	0.504	−3.1 (−4.0, −2.3)	0.246
Meat	5.8 (10.7)	1.3 (4.3)	<0.001	−3.7 (−4.5, −2.9)		−3.8 (−4.6, −3.0)	
SOL							
Fish	23.9 (15.9)	23.0 (16.0)	0.478	−1.6 (−4.4, 1.1)	0.518	−1.9 (−4.7, 0.9)	0.388
Meat	27.3 (19.1)	26.3 (16.8)	0.559	−0.4 (−3.0, 2.3)		−0.1 (−2.8, 2.6)	

^1^ Paired-samples *t*-test for comparison of individual pre- and post-intervention values within each intervention group. ^2^ Linear mixed effect model adjusted for pre-intervention score. ^3^ Linear mixed effect model adjusted for pre-intervention score and dietary compliance (amount of fish/meat consumed). Abbreviations: SOL, sleep onset latency, TIB, time in bed; WASO, wake after sleep onset.
